# Seroprevalence and Risk Factors of *Toxoplasma gondii* Infection Among High-Risk Populations in Jiangsu Province, Eastern China

**DOI:** 10.3389/fcimb.2021.783654

**Published:** 2021-10-28

**Authors:** Fanzhen Mao, Yougui Yang, Yuying Chen, Qiang Zhang, Xin Ding, Bixian Ni, Xiangzhen Xu, Xiaolin Jin, Yang Dai

**Affiliations:** ^1^ National Health Commission (NHC) Key Laboratory of Parasitic Disease Control and Prevention, Jiangsu Provincial Key Laboratory on Parasite and Vector Control Technology, Jiangsu Institute of Parasitic Diseases, Wuxi City, China; ^2^ Center for Global Health, Nanjing Medical University, Nanjing City, China

**Keywords:** Toxoplasma gondii, seroprevalence, risk factors, high-risk population, eastern China

## Abstract

*Toxoplasma gondii*, an opportunistic protozoan, infects one-third of people worldwide and could lead to serious outcomes in immunodeficient or immunocompromised populations. The present study aimed to investigate the prevalence and risk factors for *T. gondii* infection among high-risk populations in Jiangsu Province, eastern China. We conducted a cross-sectional survey among 4 categories of populations in 13 prefectures including HIV/AIDS patients, livestock breeding/processing (B/P) staff, pregnant women, and cancer patients. We detected specific immunoglobulin G and M (IgG and IgM) levels for each participant using enzyme-linked immunosorbent assay (ELISA) and asked to complete a questionnaire for each participant that covered sociodemographic information as well as the basic knowledge of attitudes toward and the practices for the prevention of toxoplasmosis. A total of 5231 participants distributed across 13 prefecture-level cities was surveyed, including 2455 males and 2776 females. Total seropositivity rate in each population category was as follows: 9.08% (HIV/AIDS patients), 11.65% (livestock B/P staff), 5.50% (pregnant women), and 12.89% (cancer patients). We detected IgM positivity in HIV/AIDS patients (0.47%, 6/1289), livestock B/P staff (0.08%, 1/1330), and cancer patients (0.46%, 6/1303) but not in pregnant women. Further, we detected IgM+IgG positivity only in cancer patients (0.31%, 4/1303). The seropositivity rate for pregnant women was significantly lower, while cancer patients were significantly higher. Higher educational levels were associated with lower seropositivity rates for *T. gondii* infection. High seropositivity rates were associated with long period of HIV infection among HIV/AIDS patients, frequent contact with livestock among livestock breeding/processing staff and male older patients among cancer patients, respectively. Analysis of practices across all participants showed that frequent contact with pets in everyday life or using the same cutting board for both raw and cooked foods leads to higher seropositivity rates. Therefore, we obtained the seroprevalence and risk factors of toxoplasmosis among high-risk populations in Jiangsu Province which could provide evidence for the implementation of control measures in the near future.

## Introduction

*Toxoplasma gondii* (*T. gondii*), an extremely successful obligate intracellular protozoan (phylum Apicomplexa, subclass Coccidia), could infect almost any nucleated-cell type of warm-blooded vertebrates, including all species of mammals and birds. It is endemic in most parts of the world, especially in warm and humid areas. More than 30% of the human population is infected, with seropositivity rates ranging from 10% to 90% (Dubey and Jones, 2008; Pappas et al., 2009; Oliveira et al., 2020; Shahrzad et al., 2020). As toxoplasmosis is an opportunistic disease, about 80% of primary infections are asymptomatic due to the effective control by the host immune system, showing up only as a transient illness featuring lymphadenopathy, fever, fatigue, and headache (Griffin and Williams, 1983; Machala et al., 2013). However, the latent infection can be activated and become a major cause of Toxoplasma-associated morbidity and mortality in immunocompromised populations, including patients with acquired immune deficiency syndrome (AIDS), pregnant women, patients with advanced cancers undergoing radiotherapy or chemotherapy, and patients undergoing immunosuppressive therapy, which could define as high-risk populations for *T. gondii* infection (Yuan et al., 2007; Gao et al., 2012; Walle et al., 2013). Clinical manifestations are diverse and range from myocarditis, ocular toxoplasmosis, encephalitis, and hydrocephalus to psychiatric disorders (Hsu et al., 2014).

The life cycle of *T. gondii* is complex, including the asexual and sexual stages. Every life stage of *T. gondii*—sporulated oocysts, tachyzoites, bradyzoites, and tissue cysts—is infectious to humans (Montoya and Liesenfeld, 2004; Schlüter et al., 2014). It has been reported that the oral transmission route is predominant in human infection in Western countries due to the ingestion of raw or uncooked food, especially meat, contaminated with *T. gondii* in any stage (Dubey and Jones, 2008; Ortega-Pacheco et al., 2013). Jiangsu is an eastern-central coastal province of the People’s Republic of China, with an average temperature of −1 to 4°C in January and 26 to 29°C in July. The annual rainfall is 800–1200 mm. The province’s environment is suitable for the transmission of *T. gondii* (Studeničováa et al., 2006). Habit of consuming raw or uncooked food was not popular in past and is growing in recent years in Jiangsu province. However, the prevalence and risk were not clear about *T. gondii* infection through oral transmission route.

To understand seroprevalence and the risk factors of *T. gondii* infection among high-risk populations of Jiangsu Province currently, we carried out a cross-sectional survey in 13 prefecture-level cities among 4 population categories: patients infected with human immunodeficiency virus (HIV) and/or suffering from AIDS, livestock breeding/processing (B/P) staff, pregnant women, and cancer patients.

## Materials And Methods

### Survey Coverage and Participants

Thirteen prefecture-level cities of Jiangsu Province—Nanjing, Wuxi, Xuzhou, Changzhou, Suzhou, Nantong, Lianyungang, Huaian, Yancheng, Yangzhou, Zhenjiang, Taizhou and Suqian—were covered in the present survey. We selected 4 categories of specific populations distributed across the 13 cities: HIV/AIDS patients, livestock B/P staff, pregnant women and cancer patients. We surveyed at least 100 participants from each specific population in each city.

### Sample Collection

We obtained a 5 ml venous-blood sample from each participant in accordance with good clinical practices, labeled it with a unique number and kept it at 4°C before transport to the laboratory. With the cooperation of the AIDS control department in each prefecture-level Centers for Diseases Control (CDC), we collected serum samples from HIV/AIDS patients during their regular checkups. All blood samples were centrifuged to remove blood cells, and serum samples were collected and stored at −80°C prior to antibody detection.

### *T. gondii*–Specific Antibody Detection

We qualitatively detected *T. gondii*–specific antibodies, including immunoglobulin G and M (IgG and IgM), in all serum samples using enzyme-linked immunosorbent assay (ELISA) commercial kits (Haitai Biological Pharmaceuticals Co. Ltd., Zhuhai, China) per manufacturer’s protocols. Briefly, we added serum samples at a dilution of 1:100 to each well of a testing plate (100 μl/well), which included the positive, negative, critical and blank controls in each kit, and incubated them at 37°C for 30 (IgG) or 60 (IgM) minutes. After washing the samples 5 times, we added 50 μl horseradish peroxidase (HRP)–labeled conjugate to each well (excluding the blank controls) and incubated them for another 30 (IgG) minutes or 60 (IgM) minutes. We then added 100 μl color reagents to each well after a final 5 washes and incubated them at 37°C for 15 minutes. We measured optical density (OD) at 450 nm with a Microplate Reader (Antobio Bio-Company, Zhengzhou, China). Each serum sample was detected with duplication. The threshold value for each testing plate was calculated by the mean of 3 critical control OD450 values, and a result equal to or greater than the threshold value was considered positive. Due to biohazard considerations, we detected serum samples from HIV/AIDS patients in the HIV testing laboratory of each prefecture-level CDC, and serum samples from the other populations were detected in the immunological laboratory of Jiangsu Institute of Parasitic Diseases.

### Questionnaire Surveys

Each participant was requested to fill out two questionnaire forms. One form asked for the participant’s demographic information, including name, gender, date of birth, ethnicity and educational level. Each specific population was asked to provide additional information on this form: HIV/AIDS patients were asked for histories of drug abuse and how long ago their diagnoses of HIV infection had been confirmed; livestock B/P staff were asked for the number of years they had done such work, which types of animals they had contact with, and the frequency of such contact; pregnant women were asked which gestational week they were in, how many pregnancies they had had, whether any of their pregnancies had been abnormal and the outcomes for such; and cancer patients were asked how long ago their diagnoses of cancer had been confirmed, as well as malignancy type. The other form requested details of the participant’s basic knowledge of toxoplasmosis (transmission routes, hazards and preventive measures), attitudes toward prevention and treatment of toxoplasmosis, and practices (frequency of contact with pets, dietary habits and whether the participant used the same cutting board for raw and cooked foods in the kitchen).

### Data Analysis

We used Epi Info software version 6 (US Centers for Disease Control and Prevention, Atlanta, Georgia, US) to establish our database, and we adopted double entry and recheck to ensure data accuracy. We used Pearson’s chi-squared test and Fisher’s exact test to investigate associations among qualitative categorical variables, and we performed all calculations with SPSS software version 17.0, (SPSS Inc., Chicago, Illinois, US). P <0.05 was considered statistically significant.

## Results

### Seroprevalences in Different Populations

**Figure 1:**
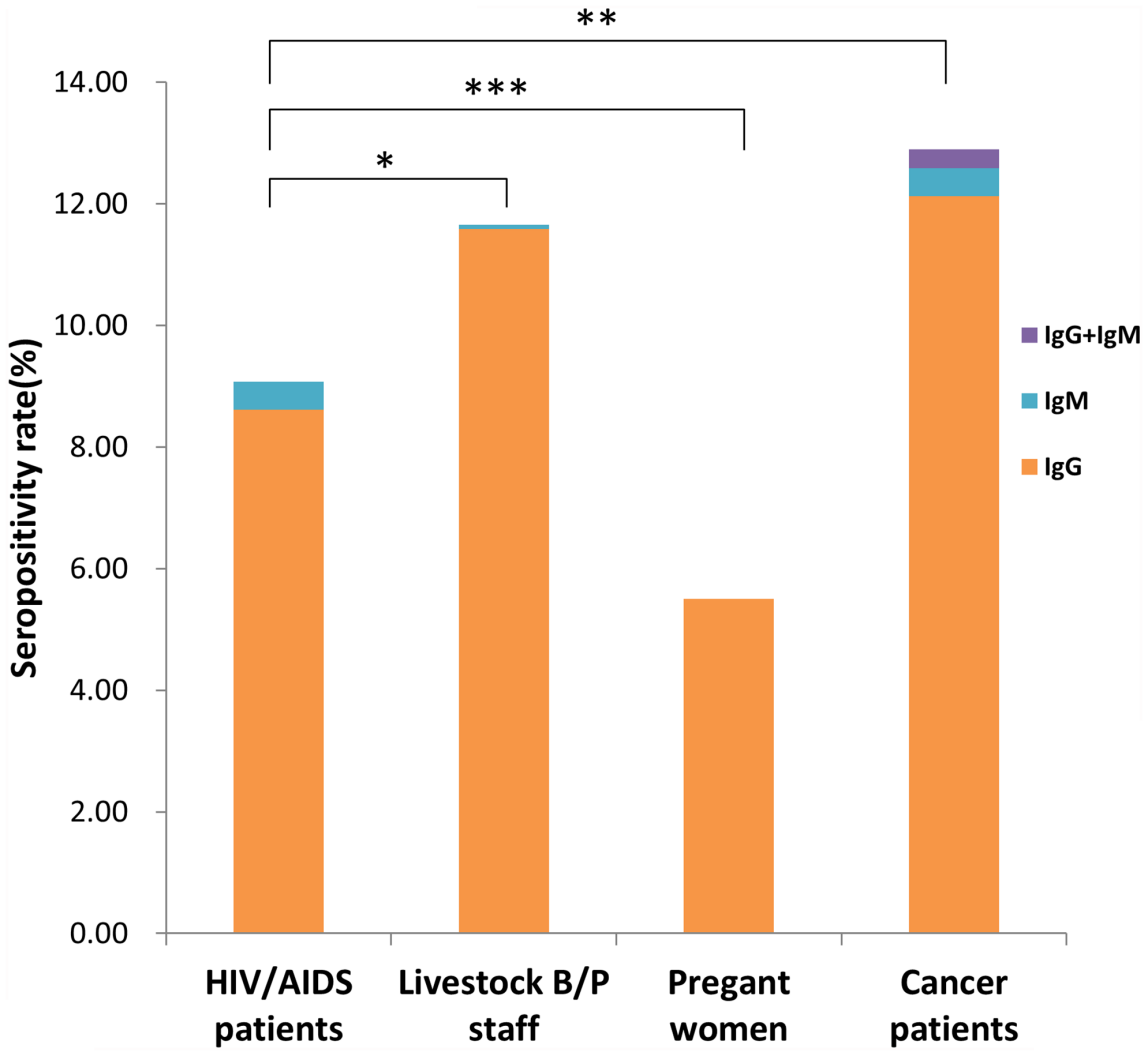
Seropositivity rate for different high-risk populations in Jiangsu Province, China. (*P < 0.05, **P < 0.01, ***P < 0.001).

We investigated a total of 5231 participants, including 2455 males and 2776 females, distributed across 13 prefecture-level cities of Jiangsu Province. Total seropositivity rates, including specific IgG, IgM and IgG+IgM positivity rates, are shown in **Figure 1**. The seropositivity rates of the 4 population categories were 9.08% (HIV/AIDS patients), 11.65% (livestock B/P staff), 5.50% (pregnant women) and 12.89% (cancer patients). There were significant differences in seropositivity rates among these categories (*x^2^* = 47.57, *P <*0.001). Seropositivity rate for pregnant women was significantly lower than that for HIV/AIDS patients, livestock B/P staff and cancer patients (*x^2^* = 12.163, 31.511 and 42.955, respectively; *P <*0.001). The seropositivity rate for HIV/AIDS patients was significantly lower than that for livestock B/P staff and cancer patients (*x^2^* = 4.672, *P* = 0.031 and *x^2^* = 9.905, *P* = 0.002, respectively). However, there was no significant difference in seropositivity rate between livestock B/P staff and cancer patients. We detected IgM positivity in HIV/AIDS patients (0.47%, 6/1289), livestock B/P staff (0.08%, 1/1330) and cancer patients (0.46%, 6/1303), but no IgM positive cases were detected in pregnant women. Furthermore, we detected IgM+IgG positivity in cancer patients only (0.31%, 4/1303).

**Figure 2:**
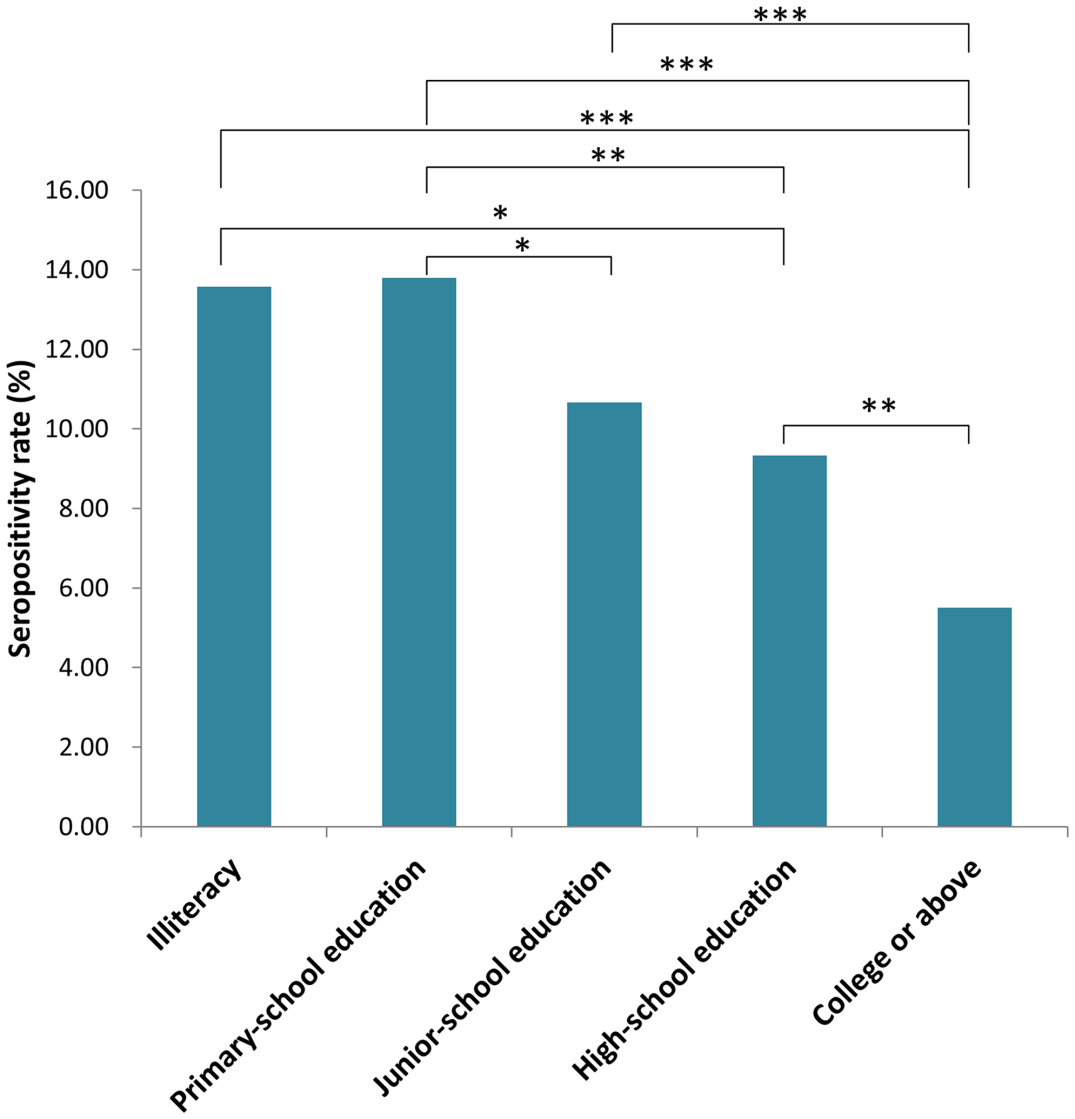
Seropositivity rate for populations with different education level in Jiangsu Province, China. (*P < 0.05, **P < 0.01, ***P < 0.001).

Participants of Han descent comprised 99.54% of subjects. The rest (0.46%) were minorities, including Miao, Hui and Manchu. There were no significant differences among participants from different ethnic groups (data not shown). Seropositivity rates for participants with different educational levels are shown in **Figure 2**. There were significant differences in rates among participants with different educational backgrounds (*x^2^* = 40.48, *P <*0.001). The seropositivity rate for participants who had gone to college or beyond was significantly lower than that for participants with no education, primary-school education or junior-school education (*x^2^* = 23.689, 36.042 and 19.772, respectively; *P <*0.001). The seropositivity rate for participants with high-school educations was also significantly lower than that for participants with no education or primary-school education (*x^2^* = 8.818, *P* = 0.003; *x^2^* = 4.967, *P* = 0.026, respectively). Furthermore, the seropositivity rate for participants with junior-school education was significantly lower than that for participants with primary-school education (*x^2^* = 5.354, *P* = 0.021).

**Figure 3:**
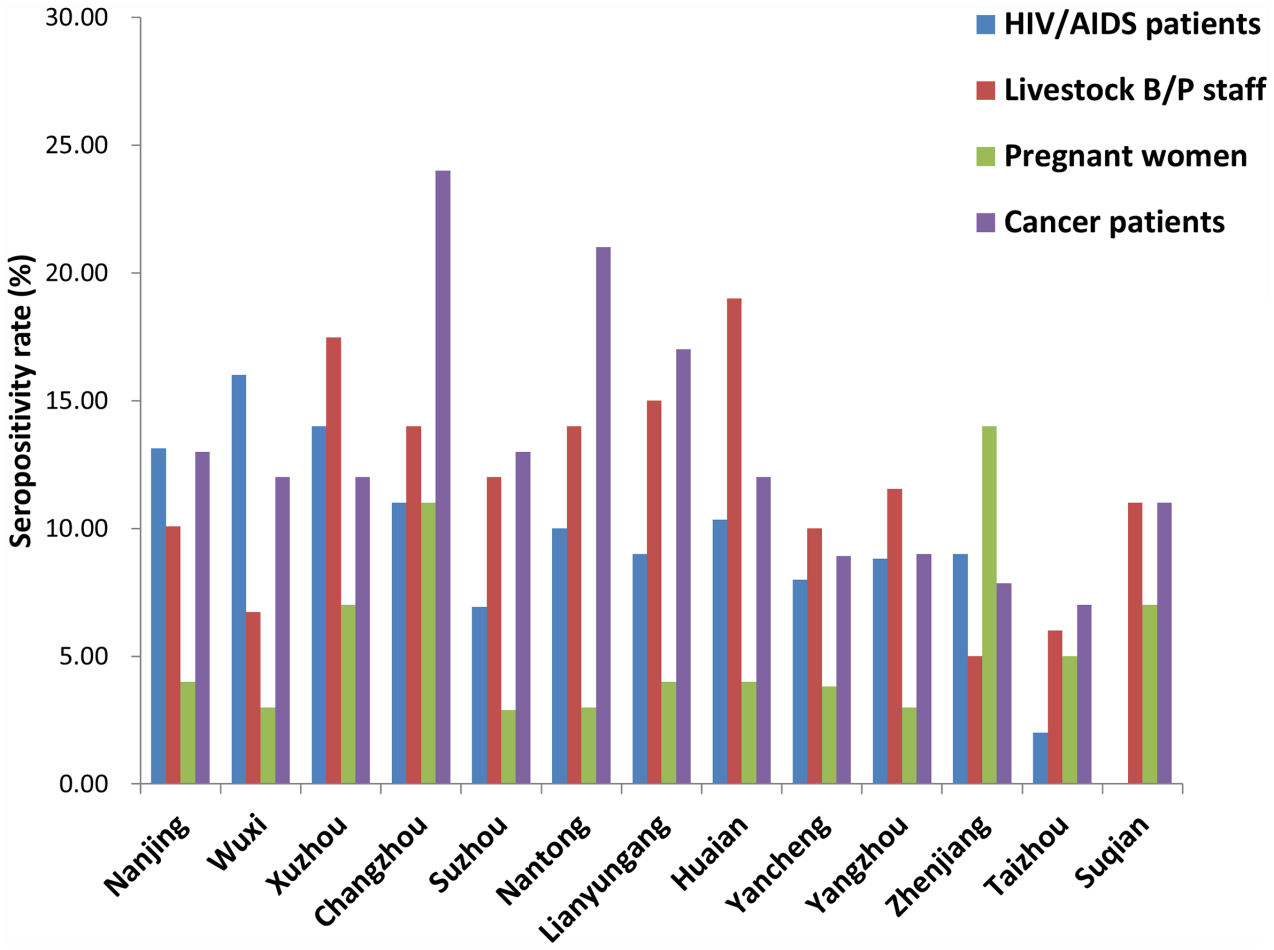
Seropositivity rate for different high-risk populations in each city of Jiangsu Province, China.

Seropositivity rates for the 4 population categories from the 13 prefecture-level cities are shown in **Figure 3**. There were significant differences among different cities for each population category (HIV/AIDS patients: *x^2^* = 28.202, *P* = 0.005; Livestock B/P staff: *x^2^* = 21.247, *P* = 0.047; pregnant women: *x^2^* = 27.485, *P* = 0.007; cancer patients: *x^2^* = 27.056, *P* = 0.008).

### Long Period of HIV Infection Was Associated With High Seropositivity Rate for HIV/AIDS Patients

**Table 1** shows the survey results for HIV/AIDS patients. We surveyed 1289 HIV/AIDS patients, including 1072 males and 217 females with ages range 2–92 years. There were no significant differences in seropositivity rates between males and females or among different age groups. Participants with histories of drug abuse accounted for 1.55% of subjects; there was no significant difference in seropositivity rate between such participants and those without histories of drug abuse. However, there were significant differences in toxoplasmosis seropositivity rate among participants who had different periods of confirmed diagnosis of HIV infection (*χ^2^* = 11.444, *P* = 0.022). Those whose diagnoses had been confirmed >3 years before had the highest seropositivity rate (15.60%).

**Table 1:** Seroprevalence of toxoplasmosis in HIV/AIDS patients.

Items	No. of participants	No. IgG-positive	No. IgM-positive	Seropositivity rate (%)	χ^2^, *P*-value
**Gender:**					
Male	1072	91	6	9.05	0.006, 0.937
Female	217	20	0	9.22	
**Age group (years):**					
0–20	41	2	0	4.88	6.871, 0.230
21–30	330	18	2	6.06	
31–40	333	30	2	9.61	
41–50	313	33	1	10.86	
51–60	178	19	1	11.24	
≥61	94	9	0	9.57	
**History of drug abuse:**					
Yes	20	2	1	15.00	0.864, 0.353
No	1269	109	5	8.98	
**Period of confirmed diagnosis of HIV infection:**					
<3 months	289	18	0	6.23	11.444, 0.022
3–6 months	301	21	0	6.98	
6–12 months	268	26	0	9.70	
1–3 years	322	33	2	10.87	
>3 years	109	13	4	15.60	
**Total:**	1289	111	6	9.08	—

### Frequent Contact With Livestock Was Associated With a High Seropositivity Rate for Livestock Breeding/Processing Staff

**Table 2** shows survey results for livestock B/P staff. We surveyed 1330 workers in total, including 606 males and 724 females whose age ranged 16–92 years. There were no significant differences in seropositivity rates between males and females or among different age groups. Of all participants, 55.56% had worked >3 years as livestock B/P staff, but there were no significant differences in seropositivity rate among workers with different lengths of tenure in livestock B/P. However, differences in seropositivity rate were significant among staff who were in contact with livestock at different frequencies (*χ^2^* = 6.728, *P* = 0.035); in addition, staff who never had contact with livestock had a low seropositivity rate (5.59%). The main types of livestock with which workers had contact included cattle, sheep and pigs, which altogether accounted for 70.68% of staff. There were significant differences in seropositivity rates among staff who were in contact with different types of livestock (*χ^2^* = 8.996, *P* = 0.029); those who had contact with sheep had the highest seropositivity rate (17.16%).

**Table 2:** Seroprevalence of toxoplasmosis in livestock breeding/processing staff.

Items	No. of participants	No. IgG-positive	No. IgM-positive	Seropositivity rate (%)	χ^2^, *P*-value
**Gender:**					
Male	606	76	0	12.54	0.851, 0.356
Female	724	78	1	10.91	
**Age group (years):**					
0–20	27	4	0	14.81	7.292, 0.200
21–30	282	22	0	7.80	
31–40	236	28	1	12.29	
41–50	425	48	0	11.29	
51–60	221	31	0	14.03	
≥61	139	21	0	15.11	
**Years working as livestock breeding/processing staff:**					
<1 year	235	30	1	13.19	1.099, 0.577
1–3 years	356	37	0	10.39	
>3 years	739	87	0	11.77	
**Frequency of contact with livestock or livestock products:**					
Every day	781	101	1	13.06	6.728, 0.035
Sometimes	406	45	0	11.08	
Never	143	8	0	5.59	
**Types of livestock with which staff had contact:**					
Cattle	321	32	0	9.97	8.996, 0.029
Sheep	134	23	0	17.16	
Pig	485	64	1	13.40	
Others	390	35	0	8.97	
**Total:**	1330	154	1	11.65	—

### No Differences in Seropositivity Rate Among Pregnant Women in Different Groups

**Table 3** shows survey results for pregnant women. We surveyed 1309 pregnant women whose age ranged 17–51 years; there were no significant differences in seropositivity rate among different age groups or among participants at different weeks of gestation. About 53.40% of participants were pregnant for the first time; there were no significant differences in seropositivity rate among participants in their first, second, third, or subsequent pregnancies. Participants with histories of abnormal pregnancy accounted for 11.46% of pregnant women surveyed; their seropositivity rate did not differ significantly from that of participants without such histories.

**Table 3:** Seroprevalence of toxoplasmosis in pregnant women.

Items	No. of participants	No. IgG-positive	No. IgM-positive	Seropositivity rate (%)	χ^2^, *P*-value
**Gender:**					
Male	0	0	0	0.00	—
Female	1309	72	0	5.50	
**Age group (years):**					
0–20	37	4	0	10.81	2.076, 0.557
21–30	991	53	0	5.35	
31–40	264	14	0	5.30	
≥41	17	1	0	5.88	
**Gestational weeks:**					
1–10	152	7	0	4.61	4.746, 0.191
11–20	573	28	0	4.89	
21–30	223	19	0	8.52	
≥31	361	18	0	4.99	
**Pregnancies:**					
1	699	48	0	6.87	5.475, 0.140
2	375	14	0	3.73	
3	137	6	0	4.38	
≥4	98	4	0	4.08	
**History of abnormal pregnancy:**					
Yes	150	8	0	5.33	0.009, 0.924
No	1159	64	0	5.52	
**Total:**	1309	72	0	5.50	—

### Older and Male Cancer Patients Had High Seropositivity Rates

**Table 4** shows survey results for cancer patients. We surveyed 1303 cancer patients, including 777 males and 526 females whose age ranged 21–105 years. There were significant differences in seropositivity rate between males and females (*χ^2^* = 11.150, *P* = 0.001) and among participants in different age groups (*χ^2^* = 11.312, *P* = 0.023). Patients whose diagnoses of cancer had been confirmed <1 year before comprised 55.91% of those surveyed; there were significant differences in seropositivity rate among participants with different periods of confirmed diagnoses (*χ^2^* = 7.842, *P* = 0.049). Participants suffered from 15 types of cancer overall, and *T. gondii* seropositivity rates for each type ranged from 0% (bladder cancer and mesothelioma) to 17.61% (esophageal cancer). There were no significant differences in seropositivity rates among participants with different types of malignancies.

**Table 4:** Seroprevalence of toxoplasmosis in cancer patients.

Items	No. of participants	No. IgG-positive	No. IgM-positive	No. IgG+IgM-positive	Seropositivity rate (%)	χ^2^, *P*-value
**Gender:**						
Male	777	112	5	3	15.44	11.150, 0.001
Female	526	46	1	1	9.13	
**Age group (years):**						
0–40	46	2	0	0	4.35	11.312, 0.023
41–50	163	9	1	2	7.36	
51–60	324	38	1	0	12.04	
61–70	489	65	2	2	14.11	
≥71	281	44	2	0	16.37	
**Period of confirmed diagnosis of cancer:**						
<1 year	728	95	5	1	13.87	7.842, 0.049
1–2 years	260	21	0	0	8.08	
2–3 years	117	19	0	1	17.09	
>3 years	198	23	1	2	13.13	
**Malignancy type:**						
Esophageal cancer	284	47	2	1	17.61	17.054, 0.253
Stomach cancer	225	28	2	1	13.78	
Liver cancer	53	6	0	0	11.32	
Lung cancer	271	36	1	1	14.02	
Breast cancer	136	13	0	1	10.29	
Cervical cancer	45	2	0	0	4.44	
Ovarian cancer	28	3	0	0	10.71	
Rectal cancer	57	3	0	0	5.26	
Colon cancer	68	6	1	0	10.29	
Pancreatic cancer	16	2	0	0	12.50	
Brain tumor	15	2	0	0	13.33	
Malignant lymphoma	46	4	0	0	8.70	
Bladder cancer	16	0	0	0	0.00	
Mesothelioma	3	0	0	0	0.00	
Widespread metastatic carcinoma	40	6	0	0	15.00	
**Total:**	1303	158	6	4	12.89	—

### Analysis of Questionnaire Survey

We distributed questionnaires to each participant and recovered 4718 valid copies, a recovery rate of 90.19%. This total included 1025 copies for HIV/AIDS patients, 1230 for livestock B/P staff, 1191 for pregnant women and 1272 for cancer patients. Awareness of toxoplasmosis hazards, transmission routes and preventative measures differed among the 4 population categories. Pregnant women had the highest awareness rate (>80%; see details in **Supplementary Table 1**). Awareness of transmission routes and awareness of preventative measures (Q2 and Q4 in **Supplementary Table 1**, respectively) were lower than for the other measures among the different populations, except for pregnant women. More than 80% of participants had a positive attitude toward prevention and treatment of toxoplasmosis, and >90% of pregnant women had such an attitude. **Table 5** shows the practices and seropositivity rates for all 4 categories. We found that 38.53% of participants had frequent contact with cats in their everyday lives, and their seropositivity rate was higher than that of participants who had only occasional contact with cats (*χ^2^* = 48.794, *P <*0.001). Only 15.54% of participants had a habit of eating raw or uncooked food; their seropositivity rate did not differ significantly from those who did not have such a habit. Finally, 58.99% of participants did not use separate cutting boards for raw and cooked foods in their kitchens; their seropositivity rate was higher than that of participants who did use separate boards (χ^2^ = 9.359, *P* = 0.002).

**Table 5:** Practices for prevention of and seropositivity rates of toxoplasmosis in the 4 population categories.

Items	Frequency	Proportion (%)	Seropositivity frequency	Seropositivityrate (%)	χ^2^, *P*-value
**Q1. Do you have frequent contact with cats in your everyday life?**					
Yes	1818	38.53	256	14.08	48.794, <0.001
No	2900	61.47	225	7.76	
**Q2. Do you have a habit of eating raw or uncooked food?**					
Yes	733	15.54	76	10.37	0.028, 0.866
No	3985	84.46	405	10.16	
**Q3. Do you use separate cutting boards for raw and cooked foods in your kitchen?**					
Yes	1935	41.01	166	8.58	9.359, 0.002
No	2783	58.99	315	11.32	

## Discussion

The present study is the first of its kind to carry out a seroepidemiological survey of toxoplasmosis among the 4 population categories in the Jiangsu Province. Our data could aid in understanding the epidemiology of toxoplasmosis among vulnerable populations and provide evidence for the implementation of control measures in the near future.

Seroprevalence of *T. gondii* infection varies by country, the area of a given country, and the community (population) surveyed (Allain et al., 1998; Torgerson and Mastroiacovo, 2013; Sandoval-Carrillo et al., 2020). Prevalence also changes according to such factors as social and cultural habits, geography, and climate (Studeničováa et al., 2006). It has been reported to exceed 60% in the tropical regions of Africa and Latin America (Fernandes et al., 2009; Pappas et al., 2009). The prevalence of *T. gondii* infection is 8.8–37.3% for pregnant women in the Indian subcontinent, as high as 40% for AIDS patients in Brazil, and approximately 8.38% for cancer patients in the Anhui Province, China (Vidal et al., 2004; Singh et al., 2014; Lin et al., 2015). Based on the infection routes, pathogenic characteristics, and adverse outcomes of the infection, we selected 4 population categories to survey. Results showed that the seropositivity rates varied among these populations. Those of cancer patients and pregnant women were, respectively, the highest and lowest among the surveyed populations. In comparison with China’s mean nationwide seroprevalence of 7.9%, as reported in 1999, HIV/AIDS patients, livestock B/P staff, and cancer patients had higher seropositivity rates, while pregnant women had a lower rate (Zhou et al., 2008).

Specific IgM antibodies are used to test patients for acute toxoplasmosis because these antibodies can be detected at high titers during the onset of the disease. Meanwhile, specific IgG antibodies could be indicative of chronic toxoplasmosis (previous or latent infection) (Bono et al., 1989; Gras et al., 2004). We obtained IgM and IgM+IgG positive cases in HIV/AIDS patients, livestock B/P staff, and cancer patients, but not in pregnant women. Data suggested that acute toxoplasmosis infection may occur in HIV/AIDS patients, livestock B/P staff, and cancer patients. However, Dhakal et al. showed that only 22% of participants who were IgM+IgG-positive had such infection (Dhakal et al., 2015). Therefore, cases of acute toxoplasmosis should be identified by a combination of clinical symptoms and further tests, such as IgG titer, specific antigen detection and etc. (Kodym et al., 2010).

As an opportunistic parasitic disease, *T. gondii* infection could cause serious harm to immunodeficient or immuno-compromised hosts, such as HIV/AIDS patients or cancer patients undergoing radiotherapy or chemotherapy (Yuan et al., 2007; Dubey and Jones, 2008). It has been reported that the seroprevalence rate of latent *T. gondii* in Ethiopia may be as high as 93.3% in HIV-infected individuals, higher than that in HIV-uninfected individuals (86.7%) (Shimelis et al., 2009). In the present study, we surveyed 1289 HIV-infected participants, whose seroprevalence rate was 9.08%. We observed no significant differences in the seroprevalence rate among participants of different genders, age groups, or histories of drug abuse. Participant selection may have led to inconsistent results, as we selected HIV-infected participants who had regular checkups (not patients hospitalized with AIDS-related illnesses). However, our results showed that long periods of confirmed diagnosis of HIV infection seemed to lead to a high *T. gondii* seroprevalence rate.

Workers who breed or process livestock have a more frequent contact with animals or animal products, leading to a higher probability of becoming infected with *T. gondii*. Previous studies have shown that the IgM-positivity rate could be as high as 38.1% in livestock/farm workers (Adesiyun et al., 2011). In the present study, we surveyed 1330 livestock B/P staff, including slaughterers, dairy workers, meat processors, and animal breeders; their overall seroprevalence rate was 11.65%. There were no significant differences in the seropositivity rate by sex, age group, or the number of years spent working in such jobs. However, participants with higher frequency of contact with livestock (every day or sometimes) showed higher seropositivity rates. Detection of *T. gondii* infection in animals or animal products has been reported in several publications. It was shown that adult sheep and lambs in the US had high seroprevalence (62.4%) and could be an important source of infection in humans (Dubey and Jones, 2008). Also, polymerase chain reaction (PCR) assay results have shown that positivity rates of *T. gondii* infection may be as high as 33.13% and 43% in the muscles of live swine and in livestock products (chicken, beef, and pork), respectively (Franco-Hernandez et al., 2016; Veronesi et al., 2017). We found that workers who had contact with sheep had the highest seropositivity rate (17.16%) of all livestock workers in our survey. The infection rate of sheep and its relationship with high seropositivity rate of staffs in present study need to be further investigated.

Pregnant women with *T. gondii* infection have a broad range of severe clinical manifestations during pregnancy (Gao et al., 2012). Previous data have shown a wide range of seroprevalence, 4–85%, in women of childbearing age and/or pregnant women from different regions of the world (Tenter et al., 2000; Pappas et al., 2009; Lobo et al., 2017). We surveyed 1309 pregnant women and found that their seropositivity rate was 5.5%, which was lower than that of the other populations surveyed. The low rate may be due to the population’s high educational background (about 70% with high-school education and beyond) and the benefits of prenatal health education. There were no significant differences in seroprevalence among pregnant women by age group, weeks of gestation, number of pregnancies, or the history of abnormal pregnancy. Further, we detected no IgM positivity of the population in present survey.

Toxoplasmosis has become a great public health concern for cancer patients undergoing radiation treatment or chemotherapy in China, as the rupture of preexistent cysts could lead to active parasitemia and other serious outcomes (Cong et al., 2015; Lin et al., 2015). Seroprevalence rates in general and in specific types of cancer have varied by study. It was reported that 23.98% of cancer inpatients in Changchun, China were IgG-positive and 2.25% IgM-positive, and that the IgG-positivity rates were significantly higher in case of nasopharyngeal carcinoma and rectal cancer than in other cancers (Yuan et al., 2007). Furthermore, 8.38% of cancer patients in Anhui, China had positive IgG antibodies, but did not show any significant difference in seropositivity rates by malignancy (Lin et al., 2015). The seropositivity rate of cancer patients was 12.89% in the present study, and we did not find significant differences in such rates by malignancy. However, we did observe significant differences by sex, age group, and the period of confirmed diagnosis of cancer: participants who were male and/or >71 years old had a higher seropositivity rate.

We designed and asked 10 questions addressing the knowledge of, attitude toward, and practices for the prevention of toxoplasmosis in our questionnaire. Knowledge results showed that pregnant women achieved high correct rates and cancer patients achieved low correct rates, which may have contributed to the seroprevalence outcomes among these populations. We also observed that knowledge correct rates about transmission and prevention of toxoplasmosis were relatively low for all populations except pregnant women. This suggested that we should place more emphasis on these factors in health education in the near future. Fortunately, we found that >80% of respondents had a positive attitude toward toxoplasmosis prevention and treatment. An analysis of practices across all participants showed that frequent contact with pets, especially cats, in everyday life and using the same cutting board for raw and cooked foods may be risk factors for toxoplasmosis and could lead to higher seropositivity rates. Previous studies have shown that the predominant route of infection for *T. gondii* is oral and that ingesting raw/uncooked food (especially meat) could be the predominant transmission route in Western countries (Dubey, 1996; Dubey and Jones, 2008). However, participants with a habit of eating raw/uncooked food did not differ significantly in their seropositivity rates from other participants in our study.

In summary, the present study showed different levels of seroprevalence for *T. gondii* infection in vulnerable populations in Jiangsu Province, Eastern China. Furthermore, frequent contact with cats and using the same cutting board for raw and cooked foods may be important risk factors for toxoplasmosis, which could provide evidence for the follow-up intervention and health promotion of *T. gondii* infection. However, there are also some limitations in this study. Firstly, only seroprevalence was applied to detect positive people and the predominant strain of *T. gondii* were unknown. Secondly, cross-sectional design restricted the causality of risk factors for *T. gondii* infection. Other populations, such as people with mental diseases, should also be investigated in the near future.

## Data Availability Statement

The original contributions presented in the study are included in the article/**Supplementary Material**. Further inquiries can be directed to the corresponding author.

## Ethics Statement

The present survey was approved by the Institutional Review Board (IRB00004221) of Jiangsu Institute of Parasitic Diseases, Wuxi, China (Approved Number: 2015011). Written informed consent to participate in this study was provided by the participants’ legal guardian/next of kin.

## Author Contributions

YD and FM conceived and designed the study. YY, XX, XD and XJ organized the survey and detected the antibodies in laboratory. YY, YC, QZ, and BN analysed the data. YD and FM drafted and revised the manuscript. All authors contributed to the article and approved the submitted version.

## Funding

The work was supported by the National Key R&D Program of China (2020YFC1200100), and the Jiangsu Provincial Project of Invigorating Health Care through Science, Technology, and Education (ZDXKA2016016). The funders had no role in the design of the study and collection, analysis, and interpretation of data and in writing the manuscript.

## Conflict of Interest

The authors declare that the research was conducted in the absence of any commercial or financial relationships that could be construed as a potential conflict of interest.

## Publisher’s Note

All claims expressed in this article are solely those of the authors and do not necessarily represent those of their affiliated organizations, or those of the publisher, the editors and the reviewers. Any product that may be evaluated in this article, or claim that may be made by its manufacturer, is not guaranteed or endorsed by the publisher.
