# Supplementary material for: Seroprevalence and Risk Factors of Toxoplasma gondii Infection Among High-Risk Populations in Jiangsu Province, Eastern China
Source: Front Cell Infect Microbiol. 2021 Oct 28;11:783654. doi: 10.3389/fcimb.2021.783654 (PMC8581562; doi:10.3389/fcimb.2021.783654)
Supplement: Supplementary file 1 [file Table_1.docx]

**Supplementary Table 1. Knowledge of and attitude toward toxoplasmosis prevention and treatment in the 4 population categories.**

| **Items** | **HIV/AIDS patients** | | | **Livestock B/P staff** | | | **Pregnant women** | | | **Cancer patients** | |
| --- | --- | --- | --- | --- | --- | --- | --- | --- | --- | --- | --- |
|  | **Frequency** | **Proportion (%)** | | **Frequency** | **Proportion (%)** | | **Frequency** | **Proportion (%)** | | **Frequency** | **Proportion (%)** |
| **Knowledge:** | | | | | | | | | | | |
| **Q1. Have you ever heard of toxoplasmosis?** | | | | | | | | | | | |
| Yes | 732 | 71.41 | 672 | | 54.63 | 1087 | | 91.27 | 569 | | 44.73 |
| No | 293 | 28.59 | 558 | | 45.37 | 104 | | 8.73 | 703 | | 55.27 |
| **Q2. Do you know how a person becomes infected with *Toxoplasma gondii*?** | | | | | | | | | | | |
| Yes | 481 | 46.93 | 532 | | 43.25 | 1007 | | 84.55 | 371 | | 29.17 |
| No | 544 | 53.07 | 698 | | 56.75 | 184 | | 15.45 | 901 | | 70.83 |
| **Q3. Do you know whether *Toxoplasma gondii* infection is hazardous to humans?** | | | | | | | | | | | |
| Yes | 898 | 87.61 | 1037 | | 84.31 | 1122 | | 94.21 | 1076 | | 84.59 |
| No | 127 | 12.39 | 193 | | 15.69 | 69 | | 5.79 | 196 | | 15.41 |
| **Q4. Do you know how to prevent infection by *Toxoplasma gondii*?** | | | | | | | | | | | |
| Yes | 472 | 46.05 | 601 | | 48.86 | 995 | | 83.54 | 418 | | 32.86 |
| No | 553 | 53.95 | 629 | | 51.14 | 196 | | 16.46 | 854 | | 67.14 |
| **Attitudes:** | | | | | | | | | | | |
| **Q5. Would you like to have cats or dogs if it presents a risk of *Toxoplasma gondii* infection?** | | | | | | | | | | | |
| Yes | 912 | 88.98 | 1096 | | 89.11 | 1149 | | 96.47 | 1096 | | 86.16 |
| No | 113 | 11.02 | 134 | | 10.89 | 42 | | 3.53 | 176 | | 13.84 |
| **Q6. Would you like to pay for the treatment of human toxoplasmosis?** | | | | | | | | | | | |
| Yes | 987 | 96.29 | 1174 | | 95.45 | 1139 | | 95.63 | 1215 | | 95.52 |
| No | 38 | 3.71 | 56 | | 4.55 | 52 | | 4.37 | 57 | | 4.48 |
| **Q7. Would you continue to have cats or dogs once your toxoplasmosis was cured?** | | | | | | | | | | | |
| Yes | 193 | 18.83 | 201 | | 16.34 | 46 | | 3.86 | 211 | | 16.59 |
| No | 832 | 81.17 | 1029 | | 83.66 | 1145 | | 96.14 | 1061 | | 83.41 |
